# The WHO Maternal Near-Miss Approach and the Maternal Severity Index Model (MSI): Tools for Assessing the Management of Severe Maternal Morbidity

**DOI:** 10.1371/journal.pone.0044129

**Published:** 2012-08-29

**Authors:** Joao Paulo Souza, Jose Guilherme Cecatti, Samira M. Haddad, Mary Angela Parpinelli, Maria Laura Costa, Leila Katz, Lale Say, Brazilian Network for Surveillance of Severe Maternal Morbidity, Elson J Almeida, Eliana M Amaral, Melania M Amorim, Carla B Andreucci, Márcia M Aquino, Maria V Bahamondes, Antonio C Barbosa Lima, Frederico Barroso, Adriana Bione, Ione R Brum, Iracema M Calderon, Rodrigo S Camargo, Felipe F Campanharo, Luiz E Carvalho, Simone A Carvalho, José G Cecatti, George N Chaves, Eduardo Cordioli, Maria L Costa, Roberto A Costa, Sergio M Costa, Francisco E Feitosa, Djacyr M Freire, Simone P Gonçalves, Everardo M Guanabara, Daniela Guimarães, Lúcio T Gurgel, Samira M Haddad, Leila Katz, Debora Leite, Moises D Lima, Gustavo Lobato, Fátima A Lotufo, Adriana G Luz, Nelson L Maia Filho, Marilia G Martins, Jacinta P Matias, Rosiane Mattar, Carlos A Menezes, Elaine C Moises, Olímpio B Moraes Filho, Joaquim L Moreira, Marcos Nakamura-Pereira, Denis J Nascimento, Maria H Ohnuma, Fernando C Oliveira, Rodolfo C Pacagnella, Cláudio S Paiva, Mary A Parpinelli, Robert C Pattinson, Liv B Paula, Jose C Peraçoli, Frederico A Peret, Cynthia D Perez, Cleire Pessoni, Alessandra Peterossi, Lucia C Pfitscher, João L Pinto e Silva, Silvana M Quintana, Ivelyne Radaci, Edilberto A Rocha Filho, Simone M Rodrigues, Roger D Rohloff, Marilza V Rudge, Gloria C Saint'ynes, Danielly S Santana, Patricia N Santos, Lale Say, Luiza E Schmaltz, Maria H Sousa, Maria R Sousa, Joäo P Souza, Fernanda G Surita, Elvira A Zanette, Vilma Zotareli

**Affiliations:** 1 UNDP/UNFPA/WHO/World Bank Special Programme of Research, Development and Research Training in Human Reproduction, Department of Reproductive Health and Research, World Health Organization, Geneva, Switzerland; 2 Department of Obstetrics and Gynecology, School of Medical Sciences, University of Campinas (UNICAMP), Campinas, Brazil; 3 Department of Obstetrics, Instituto de Medicina Integral Prof. Fernando Figueira (IMIP), Recife, Pernambuco, Brazil; University of Cape Town, South Africa

## Abstract

**Objectives:**

To validate the WHO maternal near-miss criteria and develop a benchmark tool for severe maternal morbidity assessments.

**Methods:**

In a multicenter cross-sectional study implemented in 27 referral maternity hospitals in Brazil, a one-year prospective surveillance on severe maternal morbidity and data collection was carried out. Diagnostic accuracy tests were used to assess the validity of the WHO maternal near-miss criteria. Binary logistic regression was used to model the death probability among women with severe maternal complications and benchmark the management of severe maternal morbidity.

**Results:**

Of the 82,388 women having deliveries in the participating health facilities, 9,555 women presented pregnancy-related complications, including 140 maternal deaths and 770 maternal near misses. The WHO maternal near-miss criteria were found to be accurate and highly associated with maternal deaths (Positive likelihood ratio 106.8 (95% CI 99.56–114.6)). The maternal severity index (MSI) model was developed and found to able to describe the relationship between life-threatening conditions and mortality (Area under the ROC curve: 0.951 (95% CI 0.909–0.993)).

**Conclusion:**

The identification of maternal near-miss cases using the WHO list of pregnancy-related life-threatening conditions was validated. The MSI model can be used as a tool for benchmarking the performance of health services managing women with severe maternal complications and provide case-mix adjustment.

## Introduction

An estimated 287 000 maternal deaths occurred in 2010 around the world. Despite the substantial reduction as compared to 1990, much has to be done for achieving the relevant target of the Millennium Development Goals [1]. Most of the burden of maternal deaths is carried by low-income countries, but maternal mortality is still a relevant public health problem among middle-income countries. In this context, strengthening health systems and services to provide optimal care for women during pregnancy and childbirth is crucial, particularly to those women experiencing acute pregnancy-related complications [2]–[5].

Confidential enquiries of maternal deaths have been used for many years to understand health systems and services failures in the provision of appropriate maternal health care. Based on these enquiries, lessons can be learned and used to strengthen health systems and improve quality of care [6]. Despite the positive contribution of this approach, it has limitations, particularly in low mortality settings or at the health service level, where the amount of maternal deaths is generally insufficient to provide useful information. In the last 20 years, the concept of maternal near miss has been explored in maternal health as an adjunct to maternal-death confidential enquiries. Women who nearly died but survived complications have been studied as surrogates of maternal deaths. Among other positive characteristics, maternal near-miss cases can directly inform on problems and obstacles that had to be overcome during the process of health care. Maternal near-miss audits have been considered as useful approaches to improve maternal health care [7], [8].

In 2008, the World Health Organization (WHO) has adopted a maternal near-miss definition and established standard criteria for the identification of women presenting pregnancy-related life-threatening conditions. Women surviving any of the life-threatening conditions listed in the Table 1 during pregnancy, childbirth, or postpartum are considered as maternal near-miss cases by WHO. The WHO definition enables a common ground for the implementation of maternal near-miss assessments across countries and allows international comparisons to be carried out [9].

**Table 1:** The WHO set of severity markers (life-threatening conditions) used in maternal near-miss assessments.

	Group A*	Group B*
**Cardiovascular dysfunction**	•Shock	•pH <7.1
	•Lactate >5	•Use of continuous vasoactive drugs
		•Cardiac arrest
		•Cardio-pulmonary resuscitation (CPR)
**Respiratory dysfunction**	•Acute cyanosis	•Gasping
	•Respiratory rate >40 or <6/min	•PaO2/FiO2<200 mmHg
	•Oxygen saturation <90% for ≥60 minutes	•Intubation and ventilation not related to anesthesia
		
**Renal dysfunction**	•Oliguria non responsive to fluids or diuretics	•Creatinine ≥300 mmol/l or ≥3,5 mg/dl
		•Dialysis for acute renal failure
**Coagulation/hematological dysfunction**	•Clotting failure	•Acute thrombocytopenia (<50 000 platelets)
	•Transfusion of ≥5 units of blood/red cells	
**Hepatic dysfunction**	•Jaundice in the presence of pre-eclampsia	•Bilirubin>100 mmol/l or >6,0 mg/dl
**Neurological dysfunction**	•Metabolic coma (loss of consciousness ANDthe presence of glucose and ketoacids in urine)	•Coma/loss of consciousness lasting 12 hours or more
	•Stroke	
	•Status epilepticus/Uncontrollable fits/total paralysis	
**Uterine dysfunction**	•Hysterectomy due to infection or hemorrhage	

* A glossary with relevant operational definitions is available at reference 28. Stratification of the WHO life-threatening conditions is based on the SOFA score (reference 30). Group B reflects SOFA score categories 3 and 4 (i.e. markers of greater severity).

### Validation of the Set of the WHO Criteria for Identifying Women with Life-threatening Conditions

The WHO maternal near-miss definition and identification criteria have been developed using the collective wisdom of a group of international experts gathered by WHO in an evidence-informed process [9]. A systematic review summarizing the range of previous related experiences provided the scientific basis for the development of the WHO definition and identification criteria [10]. Intensive-care prognostic and severity assessment scores, such as the APACHE II, SAPS, MODS and particularly the SOFA score, were reviewed as possible sources of severity markers for identifying women with life-threatening conditions [11]–[13]. A secondary analysis of a previous multicountry study was conducted to explore pragmatic identification criteria for maternal near misses [14]. In addition, a small preliminary study was carried for piloting and pre-validating the criteria proposed by the WHO working group [15].

In spite of the evidence-informed process that led the development of the WHO criteria for identifying women with life-threatening conditions, an actual validation of such criteria is required. The validation of such criteria depends on the similarity between maternal deaths and maternal near-miss cases. From the conceptual standpoint, near-miss cases should be as similar to maternal deaths as possible. However, the development of criteria to identify near-miss cases is challenged by the absence of a gold-standard for near-miss cases. In addition, the identification of near-miss cases is always retrospective, i.e. the woman needs to survive the life-threatening complication in order to be considered as a near-miss case. In this context and considering a set of criteria as a diagnostic test, it is assumed that a set of criteria able to accurately “identify” maternal deaths would have as false positives the maternal near-miss cases. Truly-positive cases (maternal deaths) would be similar to false positive cases (maternal near-miss cases) except the vital status [14].

### Development of a Benchmarking Tool

Populations of critically ill patients may differ in their mortality risks, depending on the severity of individual cases, the case-mix, and the quality of the therapeutic management, among other factors. Prognostic/risk-scoring systems have been used in the assessment of medical and surgical critically ill patients. Most of these systems are based on mathematical models and aim at determining the position of individual patients in the spectrum of severity. By providing a judgment-free assessment of individual risks, these tools enable neutral case-mix adjustments for groups of patients. Some of these tools also enable an assessment of health service performance in the provision of care, as they allow an estimation of expected mortality considering the specific population case-mix. By comparing the observed mortality to the expected mortality in the population, a sense of appropriateness of care can be made [11], [12], [16], [17]. This is a benchmark approach, which uses the population that generated the model as the standard for comparison [17]. Some attempts to use these generic systems in populations of severely-ill obstetric populations have been made, but the accuracy of these systems has been considered as suboptimal, possibly due to the mismatch between the reference population (general, non-obstetric) and the target population (obstetric) [18]–[22].

The WHO criteria are a package of 25 severity markers portraying a comprehensive range of life-threatening conditions. Each of these severity markers is associated with a specific relationship with mortality. In addition, women experiencing pregnancy-related complications can present more than one of these severity markers. Thus, combinations of severity markers would be associated with different mortality risks. For instance, a woman that presents postpartum hemorrhage and undergoes a hysterectomy due to uterine atony has a different risk of death as compared to a woman presenting several markers of severity denoting multiple organ dysfunctions. Similarly, depending on the case-mix, a population of women presenting life-threatening conditions may differ in its relationship with mortality in comparison to other populations of women presenting life-threatening conditions. Thus, a benchmark tool able to minimize severity bias by providing case-mix adjustment and enable comparisons to a reference population would improve the applicability of the maternal near-miss concept and enable more appropriate comparisons between populations.

### Objectives

The present analysis aims at validating the WHO criteria for identification of women with pregnancy-related life-threatening conditions and developing a benchmark tool for severe maternal morbidity assessments.

## Methods

### Ethics Statement

This project has been reviewed and approved by the National Council for Ethics in Research (CONEP, Brazilian Ministry of Health) and by the Institutional Review Boards of each site (listed below). All data was obtained from medical records and did not identify participants. The National Council for Ethics in Research and the Institutional Review Boards of each site granted a waiver of individual informed consent.

The Review Boards of the following institutions reviewed and approved this project: Maternidade Cidade Nova Dona Nazarina Daou (Manaus, AM), Maternidade Climério de Oliveira (Salvador, BA), Hospital Geral de Fortaleza (Fortaleza, CE), Hospital Geral Dr. César Cals (Fortaleza, CE), Maternidade Escola Assis Chateaubriand (Fortaleza,CE), Hospital Materno Infantil de Goiânia (Goiânia, GO), Hospital Universitário da Universidade Federal do Maranhão (São Luis, MA), Maternidade Odete Valadares (Belo Horizonte, MG), Instituto de Saúde Elídio de Almeida (Campina Grande,PB), Hospital Universitário Lauro Wanderley da Universidade Federal da Paraiba (João Pessoa, PB), Centro Integrado de Saúde Amaury de Medeiros (Recife, PE), Instituto de Medicina Integral Prof. Fernando Figueira (Recife, PE), Hospital das Clínicas da Universidade Federal de Pernanmbuco (Recife, PE), Hospital das Clínicas da Universidade Federal do Paraná (Curitiba, PR), Hospital Maternidade Fernando Magalhães (Rio de Janeiro, RJ), Instituto Fernandes Figueira (Rio de Janeiro, RJ), Hospital das Clinicas da Universidade Federal do Rio Grande do Sul (Porto Alegre, RS), Faculdade de Medicina de Botucatu da Universidade Estadual Paulista (Botucatu, SP), Hospital da Mulher da Universidade Estadual de Campinas (Campinas, SP), Hospital e Maternidade Celso Pierro da Pontifícia Universidade Católica (Campinas, SP), Hospital Israelita Albert Einstein (São Paulo, SP), Faculdade de Medicina de Jundiaí (Jundiaí, SP), Hospital das Clínicas da Faculdade de Medicina de Ribeirão Preto da Universidade de São Paulo (Ribeirão Preto, SP), Santa Casa de Limeira (Limeira, SP), Santa Casa de São Carlos (São Carlos, SP), Casa Maternal Leonor Mendes de Barros (São Paulo, SP), Hospital São Paulo da Universidade Federal de São Paulo (São Paulo, SP).

### Study Design

Methodological details of the Brazilian Network for Surveillance of Severe Maternal Morbidity Study have been published elsewhere [23], [24]. Briefly, this is a multicenter, cross-sectional study implemented in 27 Brazilian referral maternity hospitals. A convenience sampling strategy was used to build the network of health facilities. Aiming at lessening the impact of the non-random sampling and optimizing the representativeness of Brazilian referral maternity hospitals, an adequate mix of health facilities was sought: public and private health facilities, university and non-university hospitals, at least one health facility from each of the five country macro-regions. The number of health facilities was determined based on the number of maternal deaths and maternal-near miss cases required to validate the use of the WHO criteria for pregnancy-related life-threatening conditions and carry out other relevant analysis. Based on previous studies [10], [25], a total of approximately 75,000 deliveries would have to be screened in order to identify around 100 maternal deaths and 600 maternal near miss cases.

In each hospital, a research team was responsible for carrying out prospective surveillance on severe maternal morbidity. During the one-year data collection period (between July 2009 and June 2010), all women admitted to the participating health facilities were screened against the inclusion criteria by research assistants. The inclusion criteria were: presence of any of the conditions listed in the Table 1 (i.e. potentially life-threatening conditions, WHO life-threatening conditions), maternal deaths and referral to another health facility due to severe ill health. The prospective surveillance was implemented by research assistants through daily visits to obstetric wards and other relevant facilities (e.g. intensive care units and emergency rooms). During the daily visit the attending staff was contacted and the medical charts of hospitalized women were screened for the study inclusion criteria. Medical records of eligible cases were retrieved for thorough review at hospital discharge, transfer to another hospital or death. Data was collected into a previously coded form and included demographic, medical and obstetric characteristics, primary determinants of life-threatening conditions (the first complication in the chain of events), duration of hospitalization (prior to delivery, following delivery and total time), the occurrence of life-threatening conditions, maternal and perinatal outcomes and information related to the occurrence of delays in the provision of care. All severity markers (i.e. WHO life threatening conditions) present in each case were recorded. Institutional capacity was assessed using an adapted version of the hospital complexity index developed for the WHO Global Survey project [26]. The hospital complexity index was used to determine the range of services available in each of the facilities and to summarize an institution’s capacity to provide obstetric care. This index comprised eight categories reflecting the: standard of building/basic services, maternal intrapartum care and human resources; availability of general medical care, anaesthesiology, emergency obstetric services; and provision of screening tests and academic resources and clinical protocols. The original hospital complexity index was used by WHO in Latin America and Asia and adapted for use in Africa. We implemented minor adaptations to reflect the Brazilian context. An open-access, web-based, good-clinical-practice compliant database solution and study management system was used (OpenClinica, Akaza Research, LLC, 2009, Waltham, MA, USA, www.openclinica.org).

### Data Quality

Several procedures were adopted to ensure high quality data and reliable information, including preparatory meetings, site visits, close monitoring of data collection and data entry, concurrent query management, inconsistency checks, double data collection for selected medical charts, and use of a detailed manual of operation. In the preparatory meetings, study coordinators and data collectors from each site were trained in the study procedures. The study protocol, manual of operation and study forms were thoroughly reviewed; training was provided on the use of the web-based data management system. During data collection the participating hospitals received continuous support including site visits. In the site visits, the study implementation was assessed and selected medical records were checked against data entered in the data management system. In addition, the web-based data management system used in this study is compliant with Good Clinical Practice (GCP) and regulatory guidelines (e.g. 21 CFR Part 11), allowing differentiated user roles and privileges, password and user authentication security, electronic signatures, SSL encryption, de-identification of Protected Health Information (PHI). Auditing to record and monitor access and data changes aligned with a set of validation and cross checking rules were implemented as part of the online data-management. Through this comprehensive package of data quality procedures reliable and high quality data were obtained.

### Statistical Analysis

The Maternal Mortality Ratio (MMR) with 95% Confidence Intervals (CI) was determined based on the total of live births that took place in the participating hospitals during the data collection period. The study maternal mortality data was compared with the WHO maternal mortality estimates for 2010 in Brazil (together with its range of uncertainty) [1]. Sensitivity, specificity, positive and negative likelihood ratios were used to determine the accuracy of the WHO criteria in the identification of women with life-threatening conditions. Vital status at discharge was considered as the gold standard for identifying women with life-threatening conditions. The prevalence of women with life-threatening complications, maternal deaths and maternal near-miss cases was calculated. In addition, the prevalence and mortality estimates together with crude relative risks and 95% CI were calculated for each severity marker.

It was hypothesized that the number of severity markers present in each case would be correlated with mortality. The study population was categorized according with the total number of severity markers per case. In each category, the frequency of cases and mortality with 95% confidence interval was determined. The correlation between number of markers per case and mortality was determined by calculating the Pearson’s correlation coefficient. In each case, the total number of severity markers is hereby defined as the maternal severity score.

Two binary logistic regressions models were developed and tested to describe the relationship between (severe) morbidity and mortality. In order to enable this, the study population was split in two: the subpopulation “A” (used for model development), and the subpopulation “B” (used for model testing) [27]. The size of the subpopulation “B” was determined considering that the area under the Receiver Operating Characteristic curve (AUROC) would be used as an estimator of model validity. The following parameters were used in the subpopulation “B” sample size calculation: 0.05 as the probability of making a Type I error, 0.20 as the probability of making a Type II error, AUROC 0.500 as null hypothesis value, and 0.800 as the minimum expected AUROC. Based on these parameters, a total of 28 maternal deaths would be required in the subpopulation “B” for the comparison of the AUROC with the null hypothesis value. Considering the total number of maternal deaths included in the study database, 80% of the study population was randomly allocated to the population “A” and the remainder (20%) was allocated to the population “B”.

The model I consisted in a univariate analysis including only the maternal severity score (i.e. the number of WHO severity markers). The model II tested the maternal severity score, distal predictors of maternal deaths (e.g. obstetric and demographic variables, direct and indirect causes of maternal deaths) and life-threatening conditions categorized as presented in the Table 1. In the multivariate analysis (model 2), a stepwise approach was used and only predictors that contributed significantly to the model were retained (the probabilities for stepwise were set as 0.05 (entry) and 0.10 (removal)). These logistic regression models estimate the probability of maternal death based on the presence or absence of severity markers and other relevant characteristics [27]. The Hosmer-Lemeshow goodness-of-fit test was used to assess the ability of these models in adequately describing the data. The Nagelkerke R square test was used to estimate the proportion of variance in maternal mortality associated with the models’ predictors. The area under the ROC curve was determined for the Maternal Severity score and the models I and II. The best performing model was selected to generate the Maternal Severity Index (MSI), hereby defined as the estimated probability of maternal death.

PASW Statistics 18, Release Version 18.0.0 (SPSS, Inc., 2009, Chicago, IL, www.spss.com) was the main statistical package used in this analysis. MedCalc® Version 11.6.1.0 (MedCalc Software,2011, Mariakerke, Belgium, www.medcalc.org) was used for AUROC sample size calculation.

## Results

**Figure 1:**
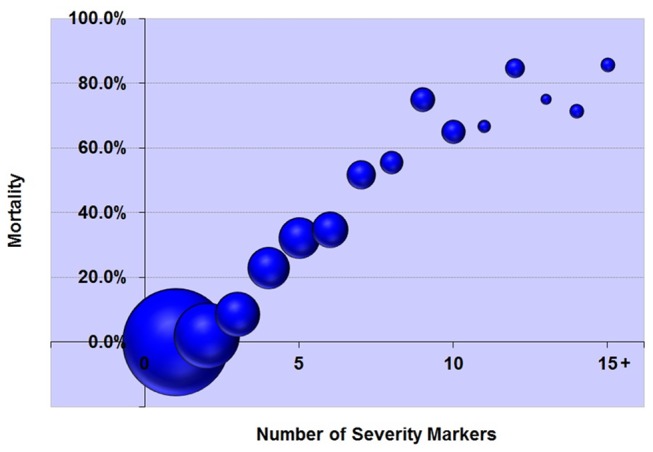
The relationship between the number of severity markers and mortality (the size of each bubble denotes the amount of cases).

A total of 82,388 women were admitted to the 27 health facilities during the one-year data collection period. These women gave birth to 82,144 born-alive infants. The study population comprised 9,555 women presenting pregnancy related complications and meeting the study inclusion criteria. Of this population with pregnancy-related complications, 910 women presented at least one of the severity markers classified as life-threatening conditions by WHO, including 140 maternal deaths and 770 survivors. The MMR in the screened population was was 170 maternal deaths per 100,000 live births (95% CI 144–201 maternal deaths per 100,000 live births). The maternal deaths included in this study represent about 8% of all maternal deaths that are estimated to occur in Brazil in 2010 (range of uncertainty: 5–13%) [1]. Table 2 shows the performance of the WHO set of criteria in two populations: all women giving birth during the data-collection period and those presenting pregnancy-related complications. All women that died presented at least one of the listed life-threatening conditions. Table 3 presents the relationship between markers of severity (WHO criteria) and maternal deaths. The prevalence of each of these life-threatening conditions ranged from 0.19 to 3.55 cases per 1000 deliveries and the condition specific mortality ranged between 12.9% and 85.0%. All life-threatening conditions were highly associated with maternal deaths, but heterogeneity was observed: 5 life-threatening conditions presented relative risks between 10 and 20, 14 presented relative risks ranging from 20 to 60, and 6 presented relative risks over 60. The amount of severity markers per case (the maternal severity score) presented a very high positive correlation with mortality as illustrated in the Figure 1 and Table 4.

**Table 2:** Accuracy of the WHO set of severity markers in the prediction of maternal deaths among all women and women with pregnancy-related complications *.

		All women		Women with complications	
		(N = 82388)		(N = 9555)	
		Maternal deaths		Maternal Deaths	
		+	−	+	−
Any WHO criterion	+	140	770	140	770
	−	0	81478	0	8645
Accuracy estimator					
Sensitivity (95% CI)		1.0 (0.97–1.0)		1.0 (0.97–1.0)	
Specificity (95% CI)		0.99 (0.99–0.99)		0.92 (0.91–0.92)	
Positive likelihood ratio (95% CI)		106.8 (99.56–114.6)		12.2 (11.4–13.1)	
Negative likelihood ratio (95% CI)		0.0		0.0	

* In this table maternal near-miss cases are the false positives.

**Table 3:** Relationship between severity markers (WHO criteria) and maternal deaths.

	Maternal Deaths				Cases presenting the severity marker per 1000 deliveries*		Mortality		Relative Risk (95% CI)		
	+		−								
**Cardiovascular dysfunction**											
Shock	+	74		176		3.01		29.60%		41.7 (30.7–56.7)	
	−	66		9239							
Cardiac arrest	+	51		13		0.77		79.69%		85 (66.8–108.1)	
	−	89		9402							
pH <7.1	+	51		22		0.88		69.86%		74.4 (57.6–96.1)	
	−	89		9393							
Lactate >5	+	24		56		0.96		30.00%		24.5 (16.7–35.8)	
	−	116		9359							
Use of continuous vasoactive drugs	+	101		143		2.93		41.39%		98.8 (69.9–139.8)	
	−	39		9272							
Cardio-pulmonary resuscitation (CPR)	+	102		18		1.44		85.00%		211 (152.3–292.4)	
	−	38		9397							
**Respiratory dysfunction**											
Acute cyanosis	+	55		68		1.48		44.72%		49.6 (37.2–66.2)	
	–	85		9347							
Gasping	+	24		13		0.44		64.86%		53.2 (39.5–71.7)	
	–	116		9402							
Respiratory rate >40 or <6/min	+	74		118		2.31		38.54%		54.7 (40.5–73.8)	
	−	66		9297							
Oxygen saturation <90% for ≥60 minutes	+	80		106		2.24		43.01%		67.2 (49.7–90.8)	
	−	60		9309							
PaO2/FiO2<200 mmHg	+	53		48		1.21		52.48%		57 (43.1–75.4)	
	−	87		9367							
Intubation and ventilation not related to anesthesia	+	123		172		3.55		41.69%		227.1 (138.6–372.1)	
	−	17		9243							
**Renal dysfunction**											
Oliguria non responsive to fluids or diuretics	+	35		55		1.08		38.89%		35.1 (25.4–48.3)	
	−	105		9360							
Creatinine ≥300 mmol/l or ≥3,5 mg/dl	+	21		77		1.18		21.43%		17 (11.2–25.9)	
	−	119		9338							
Dialysis for acute renal failure	+	24		39		0.76		38.10%		31.2 (21.7–44.8)	
	−	116		9376							
**Coagulation/hematological dysfunction**											
Clotting failure	+	33		63		1.15		34.38%		30.4 (21.7–42.5)	
	−	107		9352							
Acute thrombocytopenia (<50 000 platelets)	+	30		170		2.41		15.00%		12.8 (8.7–18.6)	
	−	110		9245							
Transfusion of ≥5 units of blood/red cells	+	50		199		2.99		20.08%		20.8 (15–28.7)	
	−	90		9216							
**Hepatic dysfunction**											
Jaundice in the presence of pre-eclampsia	+	7		22		0.35		24.14%		17.3 (8.9–33.7)	
	**−**	133		9393							
Bilirubin>100 mmol/l or >6,0 mg/dl	+	13		37		0.60		26.00%		19.5 (11.8–32)	
	−	127		9378							
**Neurological dysfunction**											
Coma/loss of consciousness >12h	+	36		29		0.78		55.38%		50.5 (37.8–67.5)	
	−	104		9386							
Metabolic coma	+	6		12		0.22		33.33%		23.7 (12.1–46.6)	
	−	134		9403							
Stroke	+	10		15		0.30		40.00%		29.3 (17.6–48.8)	
	−	130		9400							
Status epilepticus/Uncontrollable fits/total paralysis	+	5		11		0.19		31.25%		22.1 (10.5–46.6)	
	−	135		9404							
**Uterine dysfunction**											
Hysterectomy due to infection or hemorrhage	+	22		149		2.06		12.87%		10.2 (6.7–15.7)	
	−	118		9266							

* N = 82,388 deliveries.

**Table 4:** Relationship between the number of severity markers (maternal severity score) and mortality*.

Number of severity markers	Total sample (N)	Maternal deaths (n)	Mortality (95% CI)
0	8645	0	0% (0%–0%)
1	402	0	0% (0%–0.9%)
2	148	3	2% (0.7%–5.8%)
3	70	6	8.6% (4%–17.5%)
4	61	14	23% (14.2%–34.9%)
5	59	19	32.2% (21.7%–44.9%)
6	46	16	34.8% (22.7%–49.2%)
7	29	15	51.7% (34.4%–68.6%)
8	18	10	55.6% (33.7%–75.4%)
9	20	15	75% (53.1%–88.8%)
10	20	13	65% (43.3%–81.9%)
11	6	4	66.7% (30%–90.3%)
12	13	11	84.6% (57.8%–95.7%)
13	4	3	75% (30.1%–95.4%)
14	7	5	71.4% (35.9%–91.8%)
15+	7	6	85.7% (48.7%–97.4%)

* The Pearson correlation coefficient between the maternal severity score and mortality is 0.96.

The logistic regression models were developed in the subpopulation “A”, which included a total of 7,674 randomly selected cases (111 maternal deaths). The models were tested in the subpopulation “B”, which included a total of 1881 cases (29 maternal deaths). The size of each subpopulation complied with the predetermined study sample size requirements. Table 5 summarizes the performance of the models and the maternal severity score. The model II presented a better death prediction performance as evaluated by the Hosmer-Lemeshow test, Nagelkerke R^2^ test, and the percentage of maternal deaths with a model-estimated death probability greater than 50%. Based on these findings, the model II was selected to become the MSI model. Table 6 presents the covariates that were retained in the model II (i.e. the MSI model) together with the coefficients that correspond to each covariate. The death probability can be obtained using the formula presented in the Table 5. A downloadable calculator has been developed to facilitate the use of the MSI (Appendix S1).

**Table 5:** The performance of the models I and II and the maternal severity score.

	Hosmer-Lemeshow test*	Nagelkerke R^2^ test£	Percentage of maternaldeaths with a model-estimateddeath probability >50% (subpopulation “A”)	Percentage of maternaldeaths with a model-estimateddeath probability >50% (subpopulation “B”)	AUROC with 95% CI@
**Model I**	<0.001	0.629	45.9%	55.2%	0.955 (0.925–0.984)
**Model II**	0.402	0.745	66.7%	69.0%	0.954 (0.922–0.985)
**Maternal Severity Score**	N.A.	N.A.	N.A.	N.A	0.955 (0.925–0.984)

* The Hosmer-Lemeshow test indicates a poor fit if p value is less than 0.05.

£ The Nagelkerke R^2^ values the proportion of variance in maternal mortality associated with the models’ predictors. Higher R^2^ values, better model performance.

@ Area under the receiver operating characteristic curve with 95% confidence intervals calculated among women with life-threatening conditions of the subpopulation “B”.

N.A.: not applicable.

**Table 6:** The Maternal Severity Index (MSI).

Constant and covariates		Coefficients	
***Constant***		β	−7.540
*Maternal severity score*			
(x_1_)	Number of life-threatening conditions* (n)	(β_1_)	0.309
***Associated conditions***			
(x_2_)	Life-threatening conditions identified in the first 24 hours of hospital stay	(β_2_)	0.287
(x_3_)	Severe pre-eclampsia	(β_3_)	−0.579
(x_4_)	Cancer	(β_4_)	3.492
(x_5_)	Any marker of cardiovascular failure#	(β_5_)	4.209
(x_6_)	Any marker of respiratory failure£	(β_6_)	1.513
(x_7_)	Histerectomy	(β_7_)	−1.169
**Determining the MSI**			
x_1_ equals to the total number of life-threatening conditions present in the specific case and in the sequence x_2_ to x_7_, x equals 1 if the condition is present or x equals 0 if the condition is absent; then:			
Logit = β + (x_1_β_1_) + (x_2_β_2_) + (x_3_β_3_) + (x_4_β_4_) + (x_5_β_5_) + (x_6_β_6_) + (x_7_β_7_)			
MSI = e^Logit^/(1+ e^Logit^)			

* Listed in the Table 1.

# Presence of any of the following conditions: pH <7.1, Use of continuous vasoactive drugs, Cardiac arrest, Cardio-pulmonary resuscitation (CPR).

£ Presence of any of the following conditions: Gasping, PaO2/FiO2<200 mmHg, Intubation and ventilation not related to anesthesia.

## Discussion

The WHO criteria for pregnancy-related life-threatening conditions were found to be highly associated with maternal deaths. Survivors of the WHO pregnancy-related life-threatening conditions can be accurately classified as maternal near-miss cases. A severity score representing the total number of life-threatening conditions present in each case and a mathematical model describing the relationship between severity markers and maternal deaths have been developed.

Scoring systems have been used to evaluate severity and outcome of critically ill patients since many years. APACHE, SAPS, and SOFA systems are among the most used ones [11]. Some of these systems are extensively used for intensive care benchmarking and quality of care assessment [16], [17]. Notwithstanding, these systems have been developed based on general critical care populations from developed countries. In these reference populations, obstetric patients were largely omitted or underrepresented, either because pregnant women were excluded, or because maternal deaths are rare events in the countries where these systems were developed. Another issue that should be acknowledged is that the physiological changes of pregnancy affect some of the markers used by general severity scoring systems leading to overestimation of severity [18]. Also, diseases that are exclusive to this period of life (e.g. eclampsia, HELLP syndrome, acute fatty liver of pregnancy, amniotic fluid embolism), have peculiar characteristics that may not have been adequately addressed by scores designed for general populations. Owing to this, the performance of these systems in obstetric population is challenged, particularly in developing countries. Limitations are observed in the discriminatory power and the calibration of generic scoring systems when applied to obstetric population [12], [15], [19]–[22].

The maternal severity score and the MSI model, developed in this study, may contribute for a better assessment of severity of obstetric populations and enable a benchmark approach to quality of care of women experiencing severe complications related with pregnancy. As part of the strengths of this analysis, the MSI model was developed in a large multicenter study, which had an appropriate sample both in terms of number of critically ill obstetric patients and maternal deaths. The study population was large enough to allow adequate model development and testing in different subsets of the study population as methodologically recommended [27]. In the end, the MSI model was found to be robust, presenting good performance and discriminatory power. There are also two additional potential advantages that should be noted: the MSI model was developed based on the WHO criteria for pregnancy-related life-threatening conditions and the study database reflects the standard of care provided in obstetric referral hospitals from a developing country.

The WHO criteria for pregnancy-related life-threatening conditions are part of a strategy promoted by WHO for assessing and improving quality of maternal health care [8], [9], [28]. These criteria are used in the identification of maternal near-miss cases in clinical audits and other near-miss studies. Together with routine implementation, there are several near-miss research projects being currently conducted around the world using these criteria. In addition to external validation, these initiatives may favor further dissemination of the maternal near miss concept and enable the use of the MSI model as a benchmark tool.

As in other severity models, the MSI model reflects the characteristics and standards of care received by the population that provided data for its development. Brazil is the largest country in Latin America and the world’s fifth largest country (both by geographical area and population). It is an upper-middle income country which overall MMR is estimated as 56 maternal deaths per 100,000 live births in 2010 by WHO [1]. This study includes a substantial proportion of all maternal deaths that are estimated to have occurred in the country during the data-collection period (8%, range of uncertainty 5–13%). The fact that the MSI model has been developed in an obstetric population is relevant for the applicability of this tool in other populations. This is particularly important in the context of other scoring systems (e.g. the APACHE family) that were developed in generic populations of developed countries. We believe that the application of the MSI to obstetric populations of other developing countries is more direct as compared to extrapolating results from non-obstetric populations from developed countries. However, it should be noted that the standard of care provided by the participating facilities is being used as the reference for the MSI estimates. One would expect that there may be still some limitations and constraints in the quality of care provided by the participating facilities. Thus, the underlying aim of using the MSI as a benchmark is to assess the health service performance against a standard and, through interventions to improve quality of care, achieve a superior performance.

This study has some limitations that are worth noting. First, the non-random nature of the facility sampling process may have introduced some level of selection bias, potentially impairing the country representativeness of this study. On the other hand, the convenience sampling approach was realistic and made this study feasible. Precautions have been taken to maximize country representativeness. An analysis based on the intra-cluster correlation coefficients provided some evidence supporting the success of these precaution measures [29]. Second, this study is largely based on information obtained from medical records. In order to reduce the chances of recording bias, information from medical records was complemented with information obtained directly from the assisting staff (if relevant information was missing and in case of doubt). In addition, several procedures to optimize quality of data have been put in place. Third, the study population is essentially provided by referral hospitals which tend to concentrate the more severe cases: the MMR observed in this study is about three times the overall MMR estimated for the country. Another aspect that deserves noting is the relationship between the various covariates within the MSI model. The maternal severity score (i.e. the total number of severity markers present in each case) is positively correlated with maternal mortality and as the number of life-threatening conditions increase, the death probability increases. If a life-threatening condition is identified at hospital arrival or within the first 24 hours of hospital stay, there is an increase in the risk of death, possibly denoting the fact that the woman has arrived in the hospital already in a very severe condition. Cancer and a cardiovascular or respiratory failure substantially increase the death risk. Two covariates (i.e. severe pre-eclampsia and hysterectomy) have negative coefficients denoting a “protective” association within the model. At the first glance this may seem counterintuitive, but these negative coefficients have to be considered in the context of severe maternal morbidity. Our interpretation to the negative coefficient associated to pre-eclampsia is that women presenting a severe health condition due to pre-eclampsia have the potential of a better outcome as compared to women in the same level of severity having other complications. This can be due to the fact that severe pre-eclampsia tend to be a transient complication and effective strategies to manage women with pre-eclampsia exist (e.g. fetal delivery, magnesium sulfate and anti-hypertensive drugs) and may have been used in the population that provided data to this model, resulting in reduced death risks. Similarly, hysterectomy plays a game-changing role in the outcome of women with uterine-related haemorrhage and infection.

There are several potential applications to both Maternal Severity Score and MSI. A primary application is determining the level of complexity and severity of a certain obstetric population. For example, a district hospital treating a population with an average maternal severity score = 0.5 is expected to require much more material and human resources than another district hospital treating a population with a maternal severity score  = 0.1. These two hypothetical district hospitals receive two different case-mix and the maternal severity score can be used to put the health service in context and support decision making for resource allocation. Another primary application is the health impact evaluation, as part of quality of care assessment. The average MSI can provide an estimation of the expected number of maternal deaths for a selected population. For example, a hypothetical obstetric population being treated in an intensive care unit or in a high dependency facility in a tertiary hospital has an average MSI of 10%. It means that in a group of 100 women treated in this facility it would be expected the occurrence of 10 maternal deaths. If 20 maternal deaths have taken place in this population, one could conclude that there may be some opportunities being missed in this facility and a strategy to improve care is needed. The MSI allows also inter-hospital and over-time comparisons. Another possible application is in research. In a randomized controlled trial, for instance, it is worthwhile determining if both trial arms are comparable in terms of severity. Severity can functions as a major confounder: trial results can differ because of unbalances in the severity of the study populations (e.g. populations containing more severe cases tend to present worse health outcomes in comparison to populations with less severe cases). The maternal severity score and the MSI can be used for adjusting for the case-mix, through stratification or as a covariate in statistical modeling.

In summary, the maternal severity score provide an estimation of the overall severity associated with a specific women or a selected population. Similarly, the MSI provides an estimate of the death risk. To maximize usability, the Appendix S1 contains a maternal severity score and maternal severity index calculator. As a final remark, the estimates derived from the MSI model are to be used with caution. A MSI with 95% of death risk means that among 100 women with similar conditions, 95 women may die. However, the model is not able to differentiate if the specific woman is among the 5 that will survive. Thus, MSI estimates should not directly guide the management of critically ill patients.

### Conclusions

The identification of maternal near-miss cases using the WHO list of pregnancy-related life-threatening conditions is valid, as these conditions are accurately associated with maternal deaths. The MSI model adequately describes the relationship between severity markers and maternal deaths. The MSI model can be used as a tool for benchmarking, population severity assessment and case-mix adjustment. The use of the MSI model within a maternal near-miss approach has the potential of contributing to the assessment and improvement of maternal health care, particularly that required by women experiencing severe maternal morbidity. Further studies assessing the performance of the MSI model in other populations are welcome.

## Supporting Information

Appendix S1 The Maternal Severity Index (MSI) Calculator
(XLSX)
