# Correction: The WHO Maternal Near-Miss Approach and the Maternal Severity Index Model (MSI): Tools for Assessing the Management of Severe Maternal Morbidity

**DOI:** 10.1371/annotation/ca8ccfaa-71c2-4bb8-8b15-121af175a43f

**Published:** 2013-11-01

**Authors:** Joao Paulo Souza, Jose Guilherme Cecatti, Samira M. Haddad, Mary Angela Parpinelli, Maria Laura Costa, Leila Katz, Lale Say, Brazilian Network for Surveillance of Severe Maternal Morbidity, Elson J Almeida, Eliana M Amaral, Melania M Amorim, Carla B Andreucci, Márcia M Aquino, Maria V Bahamondes, Antonio C Barbosa Lima, Frederico Barroso, Adriana Bione, Ione R Brum, Iracema M Calderon, Rodrigo S Camargo, Felipe F Campanharo, Luiz E Carvalho, Simone A Carvalho, José G Cecatti, George N Chaves, Eduardo Cordioli, Maria L Costa, Roberto A Costa, Sergio M Costa, Francisco E Feitosa, Djacyr M Freire, Simone P Gonçalves, Everardo M Guanabara, Daniela Guimarães, Lúcio T Gurgel, Samira M Haddad, Leila Katz, Debora Leite, Moises D Lima, Gustavo Lobato, Fátima A Lotufo, Adriana G Luz, Nelson L Maia Filho, Marilia G Martins, Jacinta P Matias, Rosiane Mattar, Carlos A Menezes, Elaine C Moises, Olímpio B Moraes Filho, Joaquim L Moreira, Marcos Nakamura-Pereira, Denis J Nascimento, Maria H Ohnuma, Fernando C Oliveira, Rodolfo C Pacagnella, Cláudio S Paiva, Mary A Parpinelli, Robert C Pattinson, Liv B Paula, Jose C Peraçoli, Frederico A Peret, Cynthia D Perez, Cleire Pessoni, Alessandra Peterossi, Lucia C Pfitscher, João L Pinto e Silva, Silvana M Quintana, Ivelyne Radaci, Edilberto A Rocha Filho, Simone M Rodrigues, Roger D Rohloff, Marilza V Rudge, Gloria C Saint'ynes, Danielly S Santana, Patricia N Santos, Lale Say, Luiza E Schmaltz, Maria H Sousa, Maria R Sousa, Joäo P Souza, Fernanda G Surita, Elvira A Zanette, Vilma Zotareli

The Appendix S1 Maternal Severity Index Calculator is currently unavailable for reader use. The accessible file can be found here: 
